# Prevalence, social acceptance, and awareness of waterpipe smoking among dental university students: a cross sectional survey conducted in Jordan

**DOI:** 10.1186/1756-0500-7-832

**Published:** 2014-11-24

**Authors:** Suhair R Obeidat, Omar F Khabour, Karem H Alzoubi, Arwa M Mahasneh, Abdel Raheem M Bibars, Yousef S Khader, Amani Alsa’di

**Affiliations:** Department of Applied Dental Sciences, Faculty of Applied Medical Sciences, Jordan University of Science and Technology, Irbid, 22110 Jordan; Department of Medical Laboratory Sciences, Faculty of Applied Medical Sciences, Jordan University of Science and Technology, Irbid, Jordan; Department of Biology, Faculty of Sciences, Taibah University, Almadina, Saudi Arabia; Department of Clinical Pharmacy, Faculty of Pharmacy, Jordan University of Science and Technology, Irbid, Jordan; Department of Community Medicine, Faculty of Medicine, Jordan University of Science and Technology, Irbid, Jordan; Jordanian Food and Drug Administration, Irbid, Jordan

**Keywords:** Tobacco, Oral health, Awareness, Attitude

## Abstract

**Background:**

Waterpipe tobacco smoking is increasing in popularity especially among young adults. This spread could be related to limited knowledge of the negative health effects of waterpipe smoking. In this study, prevalence, social acceptance, and awareness of waterpipe smoking were examined among dental university students.

**Methods:**

This is a cross-sectional survey study, where a self-administered questionnaire was completed by a sample of dental university students in Jordan.

**Results:**

Students (n = 547) reported current tobacco use of 54.3% for males versus 11.1% for females (P <0.005). Among current smokers, 3.5% used only cigarettes (22.0% males, 2.3% females), 12.6% used only waterpipe (36.6% males, 88.6% females), and 6.9% used both (41.5% males, 9.1% females). Approximately, 70% of males and 42.5% of females who used waterpipe reported smoking mostly at a café. Nearly half of the females reported that they smoke at home in the presence of parents. Among participants, 33.3% of males and 62.5% of females reported indifferent parents’ reaction regarding their waterpipe smoking. Approximately one third of students agreed with the statement that waterpipe smoking is less harmful to oral health than cigarette smoking. About 50-70% of students agreed that waterpipe smoking causes halitosis, delays wound healing time, is associated with dental implant failure, and increases the risk of dental decay.

**Conclusions:**

In this sample, waterpipe tobacco smoking was more common than cigarette smoking among dental students, especially females. This could be an implication of social acceptance of waterpipe leading to its predominance, and thus, the gradual replacement of cigarette smoking with waterpipe smoking. Additionally, dental students’ awareness about the harms of waterpipe is not optimal, and steps are needed to ensure providing such knowledge to students.

## Background

According to the World Health Organization (WHO), tobacco cigarette smoking is considered a major contributing factor to a number of non-communicable diseases including cancers and cardiovascular and lung diseases that account for 63% of all deaths worldwide, killing about 36 million people each year [1]. Recognition of the health effects of tobacco cigarette smoking has led to a significant decline in cigarette smoking initiation and use [2]. However, the decline in cigarette tobacco use is challenged by a new form of tobacco use known as waterpipe (also known as: hookah, shisha, and arghile) [3, 4].

**Figure 1:**
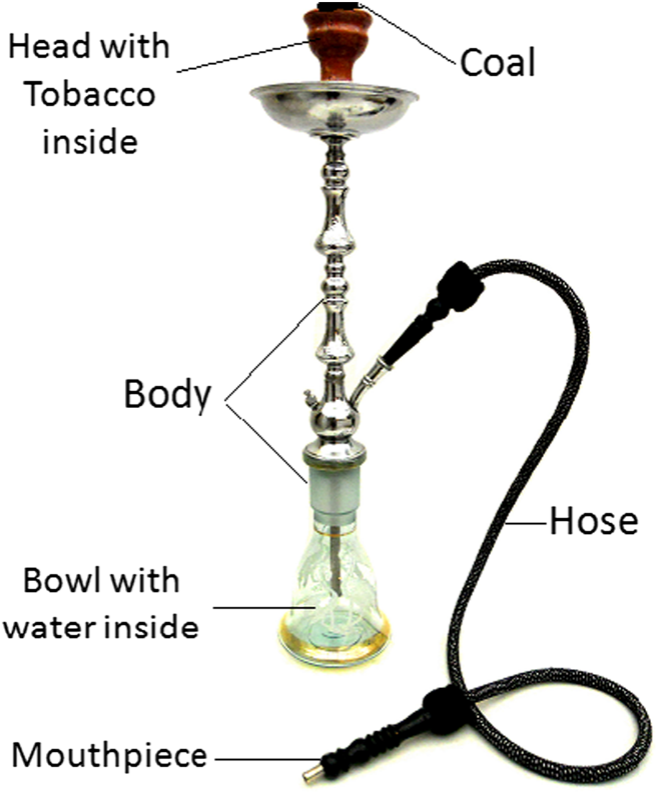
**Waterpipe showing its main parts.**

A waterpipe consists of a head, body, bowl, hose and a mouthpiece (Figure 1). The head is filled with moist sweetened tobacco, which is available in several flavors (e.g., apple, cherries, apricot). Because the tobacco does not burn in a self-sustaining manner, it is lit by placing charcoal atop the tobacco-filled head. Users draw air over the charcoal via inhaling through the mouthpiece and hose. The air, which is heated and contains charcoal combustion products, passes all the way through the tobacco producing the mainstream smoke aerosol. Smoke, then, moves through the body, bubbles through the bowl water into the hose to the mouth of the user [5].

Waterpipe tobacco smoking likely is associated with at least some of the same health risks as cigarette smoking. Aldehyde compounds found in waterpipe smoke are known to be toxic, carcinogenic and hazardous [6]. Also, a single session of waterpipe smoking produces around 46 times the tar of a single cigarette [7, 8]. The blood nicotine levels of daily waterpipe smokers are comparable to those of an individual who smokes 10 cigarettes per day [9]. Moreover, a single waterpipe use episode can increase smokers’ expired air carbon monoxide level 5 times as much as a single cigarette [10]. These data are consistent with preliminary reports linking waterpipe tobacco smoking to some systemic diseases including cancer, cardiovascular disease, decreased pulmonary function, and nicotine dependence [5, 11].

Waterpipe smoking has become prevalent among young people in developed and developing countries of the world. For example, waterpipe smoking was found to be more common than cigarette smoking with current use of 11.0% and ever use of 51.7% among medical students in London/UK [12]. A recent study that was conducted in Lebanon showed that waterpipe smoking was more common than cigarette smoking among university students [13]. In Jordan, a survey of students from four universities showed that waterpipe smoking was as prevalent as cigarette smoking with current and ever use of 30% and 56%, respectively [14]. A survey of 105,012 university students that was conducted in the U.S. showed that waterpipe is the second used method of tobacco consumption after cigarettes [15]. Similarly, high prevalence of waterpipe use was reported in countries such as Vietnam [16], Saudi Arabia [17] and Syria [18].

One reason behind the global spread of waterpipe smoking is thought to be the lack of knowledge regarding the health effects of this type of tobacco use. A substantial body of evidence demonstrates the detrimental effects of cigarette tobacco smoking on the oral health; including dental caries, dental implant failure, delayed wound healing, oral cancer, oral candidosis, hyperkeratosis, gingival and periodontal diseases, halitosis, and tooth staining [19]. However, few studies have mentioned the effects of waterpipe smoking on oral health [20]. A study conducted by Natto et al, in 2005 found that waterpipe smoking is associated with increased bone loss in periodontal diseases [21]. In addition, waterpipe smokers are at greater risk of developing dry socket following tooth extraction than non-waterpipe smokers [21, 22]. A number of studies reported that waterpipe smoking could be a predisposing factor for the development of oral cancers [20, 22–24]. Given these detrimental effects of waterpipe smoking on oral health, the overall aim of this study is to determine the prevalence, social acceptance, and knowledge about waterpipe smoking among university dental students.

## Methods

### Study design

This cross-sectional study was conducted during 2013 after obtaining approval from Institutional Review Board and research committees at Jordan University of Science and Technology (JUST). The study involved a self-administered questionnaire to a convenience sample of students from dental related fields that include Dental (D), Dental Hygiene (DH) and Dental Technology (DT) students at JUST. All enrolled Jordanian students from 2^nd^ to 5^th^ year of the included departments were invited to participate in the study. Five hundred and forty seven students agreed to participate out of 780 invited students with response rate of 70.1%. Students were approached during classes after taking permission from instructors and administrators. Students in the first university year were not targeted because they were still taking basic sciences courses, and thus are intermingled with students from other disciplines.

### Instrumentation

A self-constructed questionnaire entitled “Predominance, Knowledge and Awareness Related to Waterpipe Smoking and its Effect on Oral Health among University Students in Jordan” was developed in Arabic and used to collect data. Prior to use, the questionnaire was piloted with a sample of 20 students to test for validity and reliability. For reliability, internal consistency reliability of items of the questionnaire was carried out by calculating the Cronbach Alpha coefficient, which was equal to 0.83. This value indicates a good internal consistency of the questionnaire. The questionnaire was divided into several sections that included personal, socio-demographic variables (age, gender, university level and achievements, marital status and personal income) and smoking status (type, quantity, duration of smoking and current versus past use), knowledge about waterpipe and cigarette smoking effects on oral health.

The majority of participants took between 9 to 11 minutes to complete the questionnaire. Participation was voluntary and questionnaires were not identified by name or code to maintain anonymity and confidentiality. To participate in the study, participants were asked to read and sign a consent form. Access to data was restricted to the research team. Data entry was reviewed by random checking of 10% of entered information.

### Data processing and statistical analysis

Data were analyzed using the Statistical Package for Social Sciences (SPSS) software version 21.0 (SPSS®: Inc., Chicago, IL, USA). Numbers and percentages were produced to summarize categorical and nominal data. In addition, the Chi-square (*χ*2) test was used for comparisons among subgroups. The level of significance was set at (P <0.05).

## Results

This study included a total of 547 students. Of those, 27.6% were males, 69.8% were from the dental school, and 30.2% were from dental hygiene and dental technology departments. Table 1 shows the socio-demographic characteristics of participants according to gender. Comparable levels of academic achievement, parent education, student majors and academic year level were observed between the two groups. Current tobacco use was reported by 82 male students (54.3%) and by 44 female students (11.1%; P <0.005). The overall distribution of current smokers according to tobacco use method (i.e. cigarette versus waterpipe, Table 2) was 3.5% cigarette (22.0% of males, 2.3% females), 12.6% waterpipe (36.6% males, 88.6% females), and 6.9% used both methods (41.5% of males and 9.1% of females, Table 2).

**Table 1:** **Socio-demographic and personal characteristics of the participants according to gender**

Variable	Male N (%)	Female N (%)	Total N (%)
**Age**			
<19 years	25 (16.6)	100 (25.3)	125 (22.9)
20 years	47 (31.1)	118 (29.8)	165 (30.2)
21 years	33 (21.9)	107 (27.0)	140 (25.6)
≥ 22 years	46 (30.5)	71 (17.9)	117 (21.4)
**Students Specialty**			
Dentistry	103 (68.2)	279 (70.5)	382 (69.8)
Dental Hygiene	12 (7.9)	49 (12.4)	61 (11.2)
Dental Technology	36 (23.8)	68 (17.2)	104 (19.0)
**Academic year**			
Second year	41 (27.2)	109 (27.5)	150 (27.4)
Third year	56 (37.1)	136 (34.3)	192 (35.1)
Fourth year and higher	54 (35.8)	151 (38.1)	205 (37.5)
**Academic achievement**			
Acceptable	35 (23.3)	43 (11.2)	78 (14.6)
Good	59 (39.3)	163 (42.6)	222 (41.7)
Very good	41 (27.3)	131 (34.2)	172 (32.3)
Excellent	15 (10.0)	46 (12.0)	61 (11.4)

**Table 2:** **Smoking status of participants according to gender**

Variable	Male N (%)	Female N (%)	Total N (%)	P Value
Type of Smoking:				<0.005
Cigarettes	18 (22)	1 (2.3)	19 (15.1)	
Waterpipe	30 (36.6)	39 (88.6)	69 (54.8)	
Both cigarettes and waterpipe	34 (41.5)	4 (9.1)	38 (30.2)	
Number of waterpipe Smoking sessions				.033
Once/week	24 (37.5)	26 (60.5)	50 (46.7)	
Twice/week	14 (21.9)	9 (20.9)	23 (21.5)	
>2 times/week	26 (40.6)	8 (18.6)	34 (31.8)	
Number of years of waterpipe smoking (mean ± SD)	4.43 (2.3)	2.95 (1.7)	3.84 (2.2)	0.005
Number of cigarettes smoked				0.111
≤10 Cigarettes/Day	10 (19.6)	3 (60.0)	13 (23.2)	
10-20 Cigarettes/Day	34 (66.7)	2 (40.0)	36 (64.3)	
>20 Cigarettes/Day	7 (13.7)	0 (0.0)	7 (12.5)	
Number of years of cigarettes smoking (mean ± SD)	4.98 (3.1)	2.8 (0.8)	4.79 (3.0)	<0.005

Of those who reported current waterpipe smoking, less than half of males (40.6%) and about one fifth of females (18.6%) reported smoking more than 2 times/week (Table 2). The majority of males (83.3%) and 60.0% of females started waterpipe smoking at age of 18 years or less (Table 3). About 61.7% of males and 45.0% of females reported that they had been encouraged by friends to smoke waterpipe (Table 3). The majority of males (70%) and 42.5% of females reported smoking waterpipe most of the time at a café. About one half of females reported that they smoke at home in the presence of parents (Table 3). When they were asked for a reason for smoking waterpipe, about two thirds (61.7% of males and 59.0% of females) reported that they smoke waterpipe for pleasure, 25.0% of males and 23.1% of females reported alleviation of stress, and 6.7% of males and 23.1% of females reported “just to try” (Table 3). One third of males (33.3%) and about two thirds of females (62.5%) reported that their parents’ reactions regarding their waterpipe smoking was indifferent. About 63.3% of males and 52.5% of females reported that they had tried to quit waterpipe smoking. About half of them (42.0%) tried to quit because of the bad effects of smoking on systemic health and 21.0% because of its bad effect on oral health (Table 3).

**Table 3:** **Waterpipe smokers’ characteristics according to gender**

Questions related to the behaviors of waterpipe smokers	Male	Female	Total
	N (%)	N (%)	N (%)
**Age at starting smoking waterpipe**			
≤15 years	19 (31.6)	5 (12.5)	24 (24.0)
16-18 years	31 (51.7)	19 (47.5)	50 (50.0)
>18 years	10 (16.6)	16 (40.0)	26 (26.0)
**Having family members who smoke waterpipe**			
No one	23 (38.3)	10 (25.0)	33 (33.0)
Father	12 (20.0)	6 (15.0)	18 (18.0)
Mother	3 (5.1)	4 (10.0)	7 (7.1)
Brother	24 (40.0)	19 (47.5)	43 (43.0)
Sister	1 (1.7)	11 (27.5)	12 (12.0)
**Who encouraged you to start smoking waterpipe?**			
No one	15 (25.0)	6 (15.0)	21 (21)
Family	1 (1.7)	9 (22.5)	10 (10)
Friends	37 (61.7)	18 (45.0)	55 (55.0)
Relatives	2 (3.3)	6 (15.0)	8 (8.0)
Others	5 (8.3)	1 (2.5)	6 (6.0)
**Where do you smoke waterpipe most of the times?**			
At home in the presence of parents	13 (21.7)	21 (52.5)	34 (34)
At home when parents go out	4 (6.7)	1 (2.5)	5 (5.0)
At Waterpipe Café	42 (70.0)	17 (42.5)	59 (59.0)
Others	1 (1.7)	1 (2.5)	2 (2.0)
**With whom do you smoke waterpipe most of the times?**			
Family	5 (8.3)	15 (38.5)	20 (20.2)
Friends	49 (81.7)	21 (53.8)	70 (70.7)
Relatives	5 (8.3)	3 (7.7)	8 (8.1)
Alone	1 (1.7)	0 (0.0)	1 (1.0)
**Why do you smoke waterpipe?**			
Imitation	2 (3.3)	1 (2.6)	3 (3.0)
Alleviation of stress	15 (25.0)	9 (23.1)	24 (24.2)
For pleasure	37 (61.7)	23 (59.0)	60 (60.6)
Less harmful than cigarettes	3 (5.0)	1 (2.6)	4 (4.0)
Just to try	4 (6.7)	9 (23.1)	13 (13.1)
Other reasons	13 (21.7)	3 (7.7)	16 (16.2)
**Parents’ reactions regarding your waterpipe smoking?**			
Indifferent, I smoke Waterpipe at home when they are around	20 (33.3)	25 (62.5)	45 (45.0)
If they knew they would discipline me	21 (35.0)	8 (20.0)	29 (29.0)
Other reactions	19 (31.7)	7 (17.5)	26 (26.0)
**Have you tried to quit Waterpipe smoking?**			
Yes	38 (63.3)	21 (52.5)	59 (59.0)
No	20 (33.3)	18 (45.0)	38 (38.0)
**If yes, explain the reason:**			
I have a disease that was caused by smoking	3 (5.0)	3 (7.5)	6 (6.0)
Knowing the bad effects of smoking on systemic health	26 (43.3)	16 (40.0)	42 (42.0)
Cost	6 (10.0)	0 (0.0)	6 (6.0)
Long time needed to prepare	6 (10.0)	0 (0.0)	6 (6.0)
It’s bad effects on the oral health	12 (20.0)	9 (22.5)	21 (21.0)
Others	3 (5.0)	3 (7.5)	6 (6.0)

Table 4 shows students’ awareness and knowledge about the effects of waterpipe smoking on oral health. Approximately, one third of students agreed with the statement that waterpipe smoking is less harmful to oral health than cigarettes smoking. The majority of students agreed that waterpipe smoking transfers infectious diseases (88.1%), is a risk factor for oral cancer (82.1%), aggravates the inflammation of gum tissues (89.0%), increases the risk of dry sockets (73.4%), and causes teeth and oral tissues staining (83.4%). Lower proportions of students agreed that waterpipe smoking causes halitosis (70.3%), delays wound healing time (59.8%), is associated with dental implant failure (52.5%), and increases the risk of dental decay (64.1%). Students’ awareness and knowledge about cigarette smoking effects on oral health are shown in Table 5. The majority of students agreed that cigarette smoking is a risk factor for oral cancer, aggravates inflammation of gum tissues, increases the incidence of dry sockets, causes teeth and oral tissues staining, causes halitosis and increases the risk of dental decay (Table 5).

**Table 4:** **Students’ awareness and knowledge about the effect of Waterpipe smoking on oral health (N = 547)**

Awareness and knowledge questions	Responses N (%)								
	Agree			Disagree			Don’t know		
	Male N (%)	Female N (%)	Total N (%)	Male N (%)	Female N (%)	Total N (%)	Male N (%)	Female N (%)	Total N (%)
**Waterpipe smoking is less harmful to oral health than cigarettes smoking**	66 (44.0)	90 (22.8)	156 (28.6)	72 (48.0)	263 (66.6)	335 (61.5)	12 (8.0)	42 (10.6)	54 (9.9)
**Waterpipe smoking transfers infectious diseases**	123 (82.0)	357 (90.4)	480 (88.1)	22 (14.7)	16 (4.1)	38 (7.0)	5 (3.3)	22 (5.6)	27 (5.0)
**Waterpipe smoking is a risk factor for oral cancer**	109 (72.7)	339 (85.6)	448 (82.1)	21 (14.0)	16 (4.0)	37 (6.8)	20 (3.3)	41 (10.4)	61 (11.2)
**Waterpipe smoking may aggregate the inflammation of gum tissues**	120 (79.5)	367 (92.7)	487 (89.0)	17 (11.3)	13 (3.3)	30 (5.5)	14 (9.3)	16 (4.0)	30 (5.5)
**Waterpipe smoking increases the incidence of dry sockets**	103 (68.7)	297 (75.2)	400 (73.4)	16 (10.7)	22 (5.6)	38 (7.0)	31 (20.7)	76 (19.2)	107 (19.6)
**Waterpipe smoking causes teeth and oral tissues staining**	114 (75.5)	342 (86.4)	456 (83.4)	27 (17.9)	31 (7.8)	58 (10.6)	10 (6.6)	23 (5.8)	33 (6.0)
**Waterpipe smoking causes halitosis**	85 (56.7)	298 (75.4)	383 (70.3)	54 (36.0)	60 (15.2)	114 (20.9)	11 (7.3)	37 (9.4)	48 (8.8)
**Waterpipe smoking delays wound healing time**	84 (55.6)	243 (61.4)	327 (59.8)	26 (17.2)	48 (12.1)	74 (13.5)	41 (27.2)	105 (26.5)	146 (26.7)
**Waterpipe smoking is associated with dental implant failure**	68 (45.0)	219 (55.3)	287 (52.5)	22 (14.6)	26 (6.6)	48 (8.8)	60 (39.7)	151 (38.1)	211 (38.6)
**Waterpipe smoking increases the ones risk of dental decay**	80 (53.0)	270 (68.4)	350 (64.1)	39 (25.8)	57 (14.4)	96 (17.6)	32 (21.2)	68 (17.2)	100 (18.3)

**Table 5:** **Students’ awareness and knowledge about cigarettes smoking effects on oral health (N = 547)**

Awareness and knowledge questions	Responses N (%)								
	Agree			Disagree			Don’t know		
	Male N (%)	Female N (%)	Total N (%)	Male N (%)	Female N (%)	Total N (%)	Male N (%)	Female N (%)	Total N (%)
Cigarettes smoking is less harmful to oral health than Waterpipe smoking	54(35.8)	164(41.6)	218(40.0)	87(57.6)	189(48.0)	276(50.6)	10(6.6)	41(10.4)	51(9.4)
Cigarettes smoking transfers infectious diseases	60 (39.7)	179 (45.4)	239 (43.9)	75 (49.7)	160 (40.6)	235 (43.1)	16 (10.6)	55 (14.0)	71 (13.0)
Cigarettes smoking is a risk factor for oral cancer	122 (80.8)	345(87.6)	467 (85.7)	12 (7.9)	13 (3.3)	25 (4.6)	17 (11.3)	36 (9.1)	53 (9.7)
Cigarettes smoking may aggregate the inflammation of gum tissues	132 (87.4)	359(91.3)	491 (90.3)	9 (6.0)	13 (3.3)	22 (4.0)	10 (6.6)	21 (5.3)	31 (5.7)
Cigarettes smoking increases the incidence of dry sockets	114 (75.5)	311(78.7)	425 (77.8)	9 (6.0)	18 (4.6)	27 (4.9)	28 (18.5)	66 (16.7)	94 (17.2)
Cigarettes smoking causes teeth and oral tissues staining	134 (88.7)	372(94.2)	506 (92.7)	4 (2.6)	10 (2.5)	14 (2.6)	13 (8.6)	13 (3.3)	26 (4.8)
Cigarettes smoking causes halitosis	129 (86.6)	368 (93.4)	497 (91.5)	9 (6.0)	9 (2.3)	18 (3.3)	11 (7.4)	17 (4.3)	28 (5.2)
Cigarettes smoking delays wound healing time	96 (63.6)	255 (64.7)	351 (64.4)	22 (14.6)	42 (10.7)	64 (11.7)	33 (21.9)	97 (24.6)	130 (23.9)
Cigarettes smoking is associated with dental implant failure	84 (55.6)	236 (59.9)	320 (58.7)	17 (11.3)	20 (5.1)	37 (6.8)	49 (32.5)	138 (35.0)	187 (34.3)
Cigarettes smoking increases risk of dental decay	99 (65.6)	291 (73.9)	390 (71.6)	23 (15.2)	40 (10.2)	63 (11.6)	29 (19.2)	63 (16.0)	92 (16.9)

## Discussion

Results of this study showed that current waterpipe smoking among dental students in Jordan is higher than current cigarette smoking. This higher rate of waterpipe smoking among dental students may reflect the predominance of this type of smoking in the society and the fact that cigarette smoking is being replaced by waterpipe. Smoking waterpipe is more socially accepted as a form of modern lifestyle or prestige among the youth of the Eastern Mediterranean Region and, perhaps, the rest of the world [1, 25].

Approximately half of the students in this survey started smoking waterpipe between the ages of 16 to 18 years. This corresponds with the rise of the waterpipe epidemic in the region during the last 10 years [25]. In Lebanon, 31% of medical students started waterpipe smoking between the ages of 16 and 17 years [13]. In Jordan, 18.1 years was the mean age of waterpipe smoking initiation among university students [26].

There was a significant difference between the proportions of cigarette and waterpipe smoking among male and female dental students. Cigarette smoking was more than 10 times (22.0% vs 2.3%) as common among male compared to female dental students. Smoking waterpipe in association with cigarettes was more than four times (41.5% vs 9.1%) as common among male students compared to female students. These results can be explained by the unfavorable perception of women smoking in some Arab societies [27]. However, waterpipe smoking alone was more than two times (88.6% vs 36.6%) as common among female compared to male dental students, which can be explained by the fact that waterpipe smoking is well-tolerated and socially accepted among women [28]. This is consistent with data from Syria, Lebanon, and other Arab countries, which showed an increase in waterpipe use compared to cigarettes [13, 18, 29–31]. For example, Jradi et al [13] found a significant difference in the percentages of waterpipe, but not cigarette, smoking percentages according to gender of medical students in Lebanon, where females were more likely to smoke waterpipe than males [13]. However, in Syria, male medical students were more likely to smoke cigarettes as well as waterpipe than females [18].

The majority of dental students surveyed in this study (61.5%) reported that waterpipe smoking was more harmful to oral health than cigarette smoking, despite their smoking behavior as revealed in the results. The majority of the participants were aware that waterpipe smoking is a risk factor for oral cancer, periodontal diseases, dry sockets, and teeth and oral tissues staining. Although dental students have a good knowledge of the association between waterpipe smoking and oral health, the proportions of waterpipe smoking among students were high. Relatively lower proportions of students knew that waterpipe is associated with delayed wound healing time, dental implant failure, and dental decay. This finding may reflect the costs associated with failing to address the harmful effects of different forms of tobacco use including waterpipe when developing academic curricula. Therefore, faculties of Dentistry and Applied Medical Sciences should provide an opportunity to increase the knowledge and awareness of the risks of all methods of tobacco smoking on oral health among their dental, dental hygiene, and dental technology students. These faculties should consider adopting tobacco control programs and policies to fight against all methods of tobacco use, particularly waterpipe, among students as they are the future dentists and health educators.

We recognize that the current study has the following limitations. First, we depended on students’ self-reporting of their knowledge and practice of waterpipe smoking, which may not necessarily reflect their actual behaviors. Second, we surveyed students at only one university in Jordan. On the other hand, this study is important as it highlights the prevalence of waterpipe smoking among university students, an educated population, and the level of their health awareness about its damaging effects on oral tissues. Limited information was found on the Jordanian college students’ knowledge about waterpipe effects on oral health. The findings of this study could help in providing data to establish and develop educational and public health programs and interventions to fight this form of tobacco smoking.

## Conclusions

Current waterpipe smoking was higher than cigarette smoking among dental students especially females. This could reflect the social acceptance of waterpipe leading to its predominance, and thus, the gradual replacement of cigarette smoking by waterpipe smoking. Additionally, dental students’ awareness about the harms of waterpipe was not optimal, which highlights the shortage of academic curricula in addressing the harms of waterpipe, and the need for providing such knowledge to students.
